# p-Type hydrogen sensing with Al- and V-doped TiO_2_ nanostructures

**DOI:** 10.1186/1556-276X-8-25

**Published:** 2013-01-12

**Authors:** Zhaohui Li, Dongyan Ding, Congqin Ning

**Affiliations:** 1 Institute of Microelectronic Materials and Technology, State Key Laboratory of Metal Matrix Composites, School of Materials Science and Engineering, Shanghai Jiao Tong University, Shanghai, 200240, China; 2 State Key Laboratory of High Performance Ceramics and Superfine Microstructure, Shanghai Institute of Ceramics, Chinese Academy of Sciences, Shanghai, 200050, China

**Keywords:** TiO_2_, Nanostructures, Doping, Hydrogen sensor, p- type

## Abstract

Doping with other elements is one of the efficient ways to modify the physical and chemical properties of TiO_2_ nanomaterials. In the present work, anatase TiO_2_ nanofilms doped with Al and V elements were fabricated through anodic oxidation of Ti6Al4V alloy and further annealing treatment. Hydrogen sensing behavior of the crystallized Ti-Al-V-O nanofilms at various working temperatures was investigated through exposure to 1,000 ppm H_2_. Different from n-type hydrogen sensing characteristics of undoped TiO_2_ nanotubes, the Al- and V-doped nanofilms presented a p-type hydrogen sensing behavior by showing increased resistance upon exposure to the hydrogen-containing atmosphere. The Ti-Al-V-O nanofilm annealed at 450°C was mainly composed of anatase phase, which was sensitive to hydrogen-containing atmosphere only at elevated temperatures. Annealing of the Ti-Al-V-O nanofilm at 550°C could increase the content of anatase phase in the oxide nanofilm and thus resulted in a good sensitivity and resistance recovery at both room temperature and elevated temperatures. The TiO_2_ nanofilms doped with Al and V elements shows great potential for use as a robust semiconducting hydrogen sensor.

## Background

As a clean and renewable energy carrier, hydrogen has wide applications in the industry such as chemical production and fuel cell, as well as high-energy fuel [1]. For these applications, a robust and reliable hydrogen sensor is needed to detect a leakage during storage and transportation. Furthermore, the hydrogen sensor should also work at elevated temperatures.

To meet these targets, various kinds of hydrogen sensors based on MOSFET, catalytic combustion, electrochemical reaction, Pd metals, and semiconducting metal oxides have been reported [2-8]. As one of the important semiconducting metal oxides, titania oxide has been reported to be sensitive to hydrogen atmosphere. In the form of dense film, traditional TiO_2_ sensors usually have a higher operating temperature (between 200°C and 500°C), which limits a wide application of dense TiO_2_ film sensors [9-11]. To improve the hydrogen sensing properties of dense TiO_2_ films, doping of TiO_2_ oxides with groups III or V elements has been reported. Such a doping was found to promote chemical reactions by reducing the activation energy between the film surface and the target gas, which enhance the response and selectivity and finally reduce the maximum operating temperature of the hydrogen sensors [12-14].

To further improve the hydrogen sensing properties of traditional TiO_2_ oxides, anatase TiO_2_ nanotube arrays have been fabricated through anodization of pure Ti metals and further annealing treatment [15,16]. Hydrogen sensors made up of these undoped anatase nanotubes were usually sensitive to hydrogen-containing atmosphere by showing a decreased resistance upon exposure to the reductive atmosphere at either room temperature or elevated temperatures [17-19]. Such a resistance decrease in reductive atmosphere was a typical n-type hydrogen sensing behavior.

Ti6Al4V alloy is one of the important Ti alloys due to its excellent comprehensive properties and wide application in both industry and medical occasions [20]. As reported by Macak et al. [21], Al- and V-doped titanium oxide films could grow on the alloy substrate after surface anodization of Ti6Al4V alloy. Li et al. found that anodic Ti-Al-V-O nanofilms had good thermal stability and biocompatibility [22]. The doping engineering was expected to change the semiconducting properties of the TiO_2_ oxide.

To date, rare work has been reported on the semiconducting and hydrogen sensing properties of Al- and V-doped TiO_2_ nanofilms. Thus, in the present work, Ti-Al-V-O oxide nanofilms were fabricated for a first principle simulation and hydrogen sensing evaluation. It was shown that the Al- and V-doped TiO_2_ nanofilms could demonstrate a p-type hydrogen sensing behavior at room temperature and elevated temperatures.

## Methods

### Material and film fabrication

Ti6Al4V alloy plate in as-cast states was used as the anodic substrate. Plate sample with a size of 10 × 10 × 1 mm was grinded and polished with emery papers and then ultrasonically cleaned with absolute alcohol. Finally, they were rinsed with deionized water and dried in N_2_ stream. Electrochemical anodization was carried out with a DC voltage stabilizer. All of the samples were fabricated at 15 V (for 1.5 h) in electrolytes of 1 M NaH_2_PO_4_ containing 0.5 wt.% HF. The as-anodized samples were annealed at either 450°C or 550°C for 1 h in air to obtain crystallized nanofilms. Nanofilm sensors were fabricated using circular Pt electrodes and conductive wires for PCB assembly. Detailed sensor fabrication process can be found in our previous work [23].

### Characterization of nanostructure films

Surfaces of the above as-anodized and as-annealed samples were characterized with a scanning electron microscope (SEM; FEI SIRION 200, Hillsboro, OR, USA) equipped with energy dispersive X-ray analysis (EDXA; OXFORD INCA, Fremont, CA, USA). Surface compositions of the nanofilms were characterized with X-ray photoelectron spectroscopy (XPS; ESCALAB 250, Thermo VG Scientific, West Sussex, UK). The phase structures of the as-annealed samples were characterized with X-ray diffraction (XRD; D/max 2550 V, Rigaku, Tokyo, Japan). Grazing incident diffraction with an incident angle of 1° was carried out during the XRD testing.

### Testing of hydrogen sensors

The nanofilm sensors were tested in alternating atmospheres of air and 1,000 ppm H_2_ at temperatures ranging from 25°C to 300°C. A Keithley 2700 multimeter (Cleveland, OH, USA) was used to test the resistance of the nanofilm sensor during the hydrogen sensing experiments.

## Results

**Figure 1:**
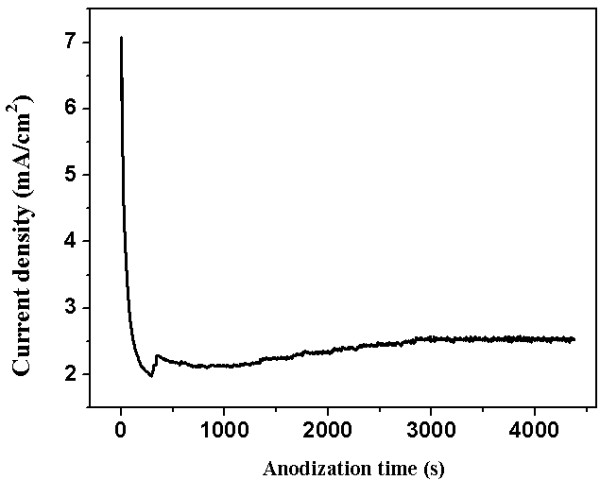
Current density vs. time curve of the anodization process.

Ti-Al-V-O oxide nanofilms formed during the anodization process. Figure 1 shows the anodization current transients (*I-t* curves) recorded at the constant anodization voltage of 15 V. The anodization current decreased rapidly from 7 to 2 mA, which corresponded to the formation of a barrier oxide at the alloy surface. At the stage of current increase to a peak value of 2.4 mA, the pores of oxide film grew randomly. After the peak point, the current decreased to reach a nearly steady-state value indicating that self-assembled oxide nanofilm could be grown on the alloy substrate [7].

**Figure 2:**
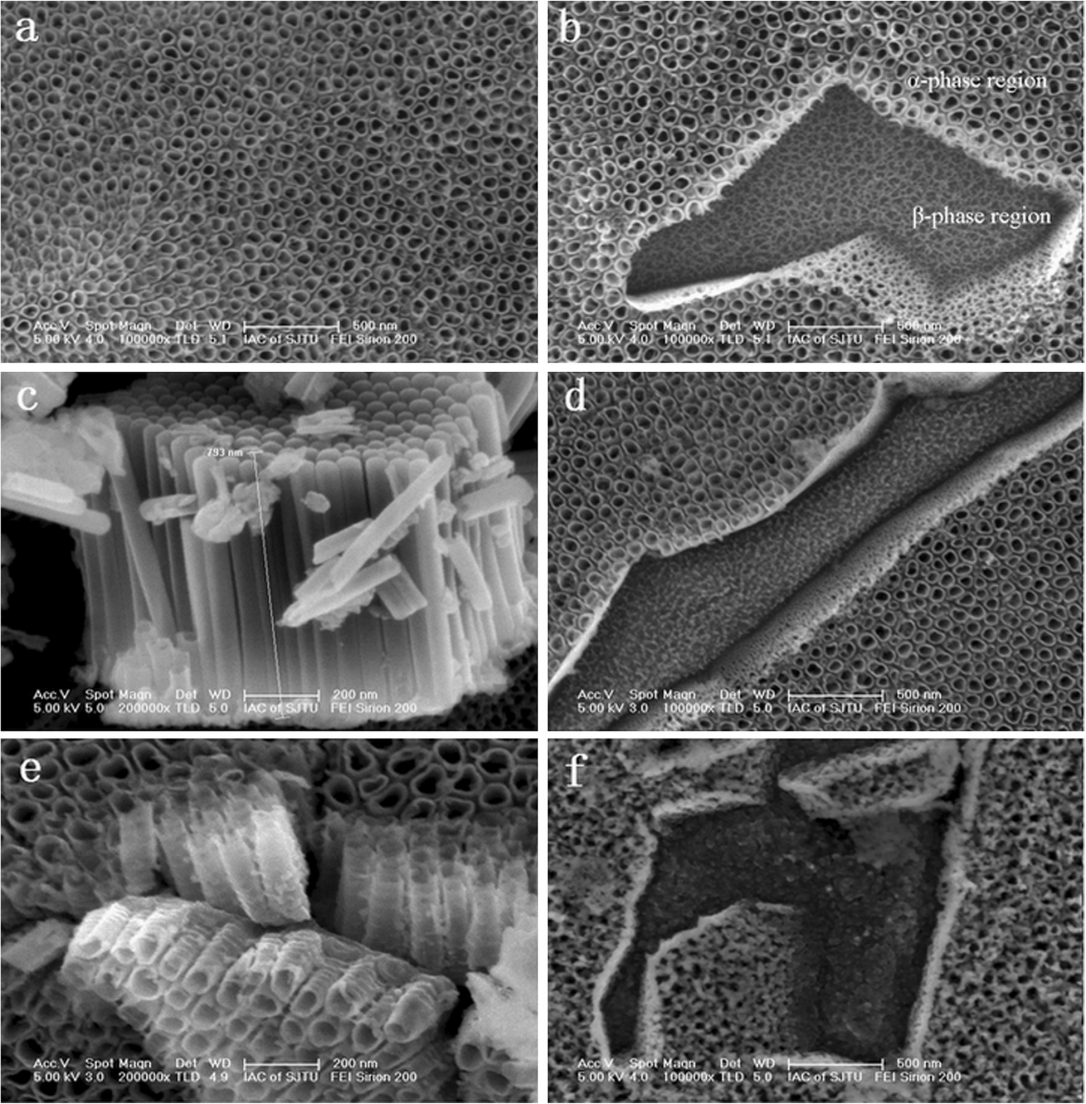
**SEM images of the oxide nanofilms before and after annealing. (a)** Top view of as-anodized sample, **(b)** α- and β-phase regions of as-anodized sample, **(c)** cross-sectional view of the Ti-A-V-O nanotubes, **(d)** top view of oxide nanofilms annealed at 450°C, **(e)** cross-sectional view of oxide nanofilms annealed at 450°C, and **(f)** top view of oxide nanofilms annealed at 550°C.

Original Ti6Al4V alloy consisted of two different phases (α and β). The major phase was α phase. Figure 2a shows the surface morphology and cross-sectional image of the oxide nanofilms grown on the Ti6Al4V substrate. The oxide nanofilms consisted of two kinds of nanostructures, i.e., nanotubes grown at the α-phase region and inhomogeneous nanopores grown at the β-phase region [22]. Average inner diameter of the nanotubes grown at the α-phase region was 65 nm, and average length of the nanotubes was around 800 nm (Figure 2c).

Figure 2d,f presents the surface morphologies of the as-annealed oxide nanofilms. In comparison with the anodic oxide nanofilms (Figure 2a,b), surface morphology of the oxide nanofilm annealed at 450°C did not change (Figure 2d,e). This suggests that both the nanotube arrays and the nanopores could bear the above annealing temperature. After annealing at 550°C, noticeable structural change in the oxide nanotubes was found. As shown in Figure 2f, the top ends of the nanotubes collapsed although the nanotubular structures could be still observed and the nanopores at the β-phase region totally collapsed and transformed to a powder-like sintering compact. Obviously, both the nanotubes and nanopores of the oxide nanofilms could demonstrate different thermal stability.

Our EDXA analysis (Table 1) of the anodic and as-annealed oxide nanofilms revealed that the oxide nanofilms consisted of four elements, i.e., Ti, Al, V, and O. It was obvious that the element content was different at different phase regions. The Ti and V elements were rich at the β-phase regions. After annealing, the weight percentage of the Ti, Al, and V elements in the oxide nanofilms decreased while the weight percentage of the O element increased.

**Table 1:** Element content of the oxide nanofilms before and after annealing at 450°C and 550°C

**Tested area**	**Oxide nanofilm condition**	**Element (at.%)**			
		**Ti**	**Al**	**V**	**O**
α-Phase region	After anodization	61.45	6.52	2.68	29.35
	Annealed at 450°C	26.90	3.39	0.87	68.83
	Annealed at 550°C	23.09	2.96	0.61	73.34
β-Phase region	After anodization	65.35	6.94	3.88	23.83
	Annealed at 450°C	44.40	4.79	2.15	48.66
	Annealed at 550°C	32.76	3.60	1.50	62.15

**Figure 3:**
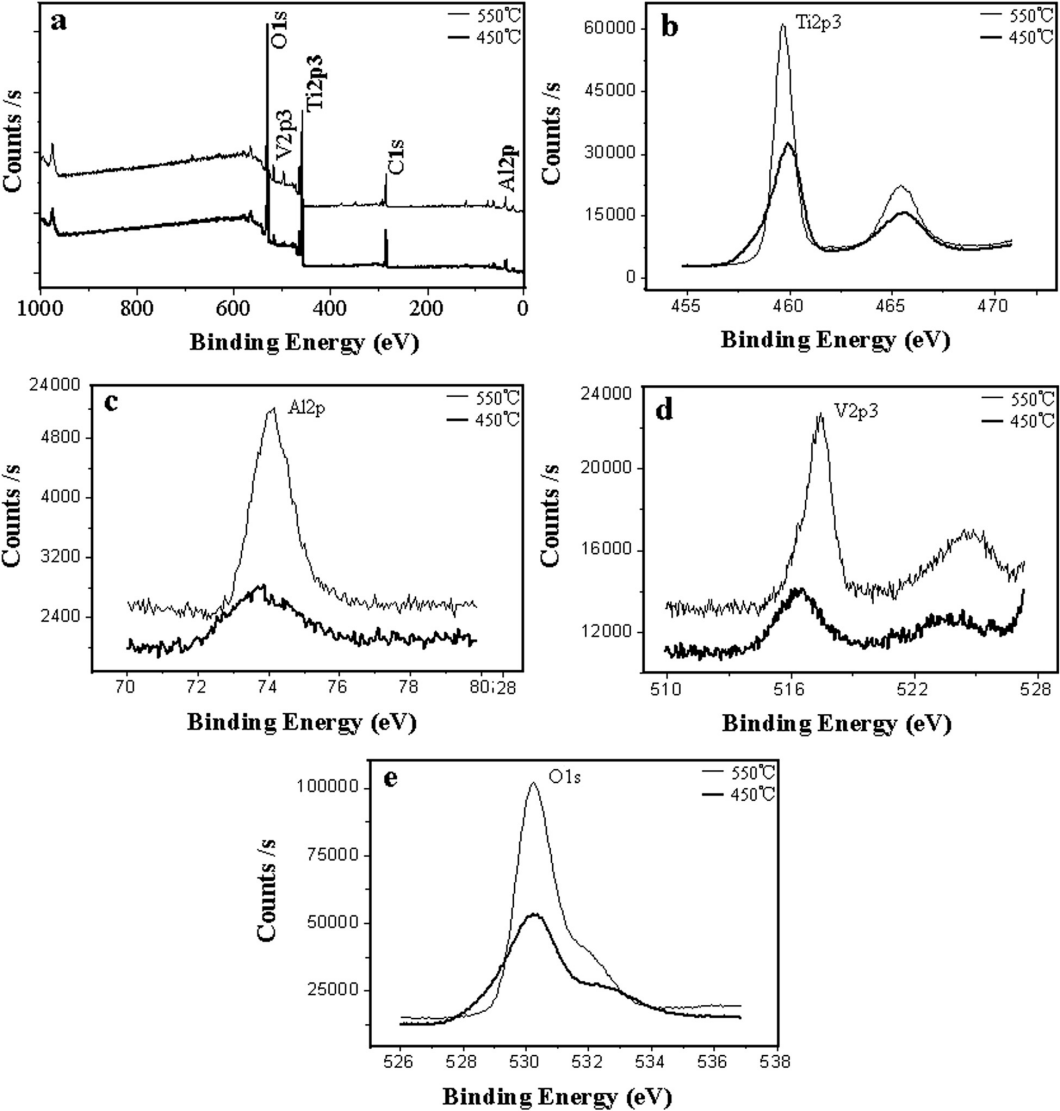
**XPS analyses of the Ti-Al-V-O oxide nanofilms annealed at different temperatures. (a)** Deconvolution of survey spectrum and **(b)** Ti 2*p*_3_, **(c)** Al 2*p*, **(d)** V 2*p*_3_, **(e)** O 1*s* scan curves.

XPS experiments were conducted to obtain more accurate surface compositions of the Ti-Al-V-O nanofilms. For the XPS spectral deconvolution (Figure 3a) of annealed oxide nanofilms, peaks corresponding to Ti, Al, V, O, and C elements were identified. The carbon peak may originate from absorbed organic groups or molecules. Figure 3b,c,d,e presents Ti 2*p*_3_, Al 2*p*, V 2*p*_3_, and O 1*s* scan patterns of the original surface of the as-annealed oxide nanofilms, respectively. At the top surface of the oxide nanofilm annealed at 450°C, the average atomic percentage of the Ti, Al, V, and O elements was 16.73%, 8.84%, 3.25%, and 71.18%, respectively. At the top surface of the oxide nanofilm annealed at 550°C, the average atomic percentage of the Ti, Al, V, and O elements was 17.14%, 5.27%, 2.13%, and 73.46%, respectively.

**Figure 4:**
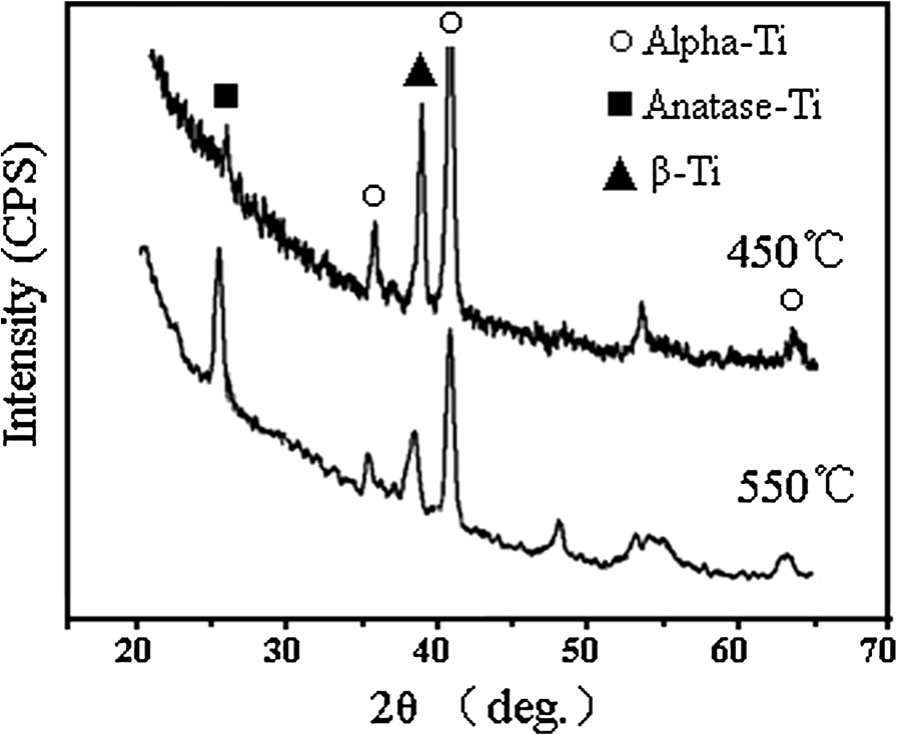
XRD pattern of the oxide nanofilms annealed at different temperatures.

Figure 4 shows the XRD patterns of the oxide nanofilms annealed at 450°C and 550°C. The diffraction peak at 25° corresponded to anatase TiO_2_. It can be found that the increase of annealing temperature from 450°C to 550°C could result in an enhanced formation of anatase TiO_2_ phase by showing a higher diffraction peak at 25°. In comparison with the traditional crystallization temperature (450°C) of undoped TiO_2_ nanotubes [7,17], the Al- and V-doped nanofilms almost had the same crystallization temperature. Obviously, the doping with Al and V elements did not significantly affect the amorphous-to-anatase phase transformation of the anodic oxide.

Hydrogen sensing properties of the oxide nanofilms were tested with an operating temperature ranging from 25°C to 300°C. The resistance of the Ti-Al-V-O nanofilm sensors tested in the hydrogen atmosphere was recorded. The response (△*R*/*R*_0_) of the nanofilm sensor is defined as follows:

(1) $$△\frac{R}{R_{0}}=\frac{R–R_{0}}{R_{0}}\times 100\%$$

where *R*_0_ is the original resistance of the sensor before exposure to the hydrogen-containing atmosphere, and *R* is the sensor resistance after exposure to or removal of the hydrogen-containing atmosphere.

**Figure 5:**
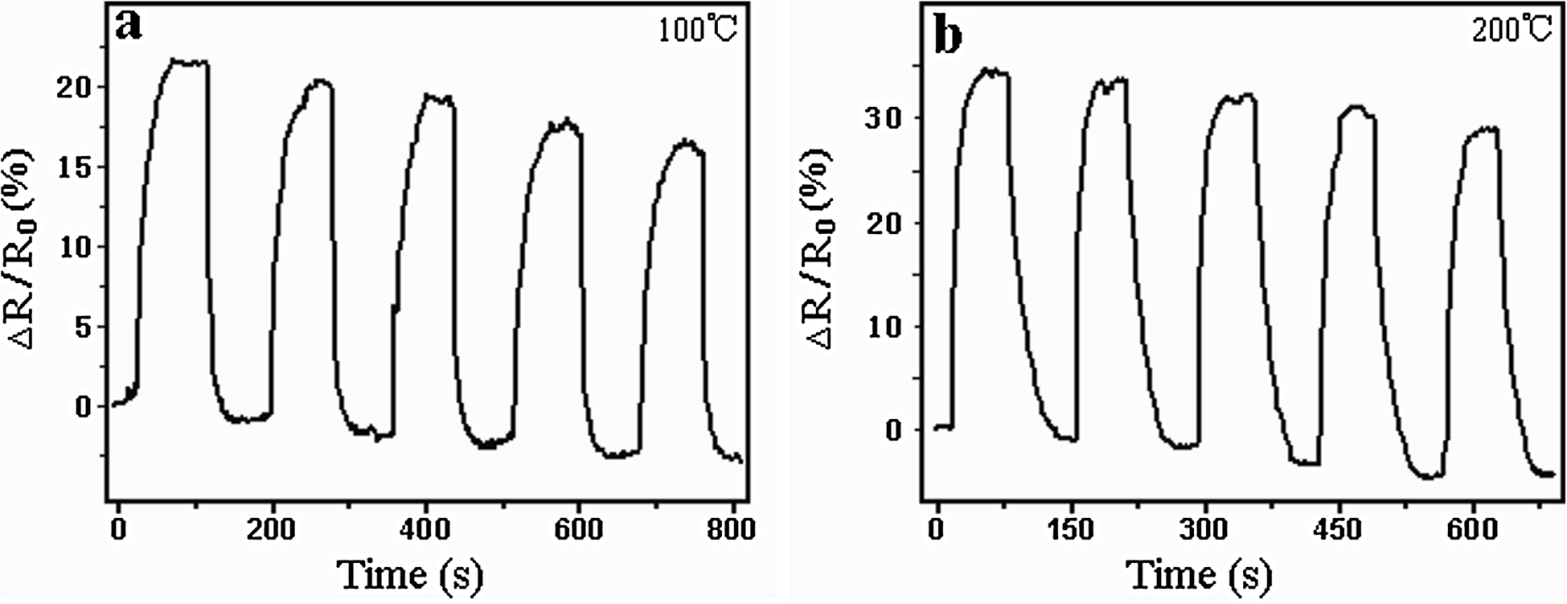
**Response curves of oxide nanofilms annealed at 450°C.** The operating temperatures were (**a**) 100°C and (**b**) 200°C.

At room temperature, the oxide nanofilm annealed at 450°C was found to have no sensitivity for the 1,000 ppm H_2_ atmosphere. Only at elevated temperatures could it demonstrate a hydrogen sensing capability. Figure 5 presents the response curve of the oxide nanofilm tested at 100°C and 200°C. The saturation response of the nanofilm sensor increased with the increase of the operating temperature. The sensor resistance increased in the presence of 1,000 ppm H_2_ and recovered in air. At 100°C, a 56% change in sensor resistance was found. At 200°C, a 77% change in sensor resistance was found. In comparison with the longer response time (about 50 s) at 100°C, the response time was reduced to 26 s at 200°C. The above facts revealed that the increase of operating temperature helped to enhance the hydrogen sensing performance of the Ti-Al-V-O nanofilm sensors.

**Figure 6:**
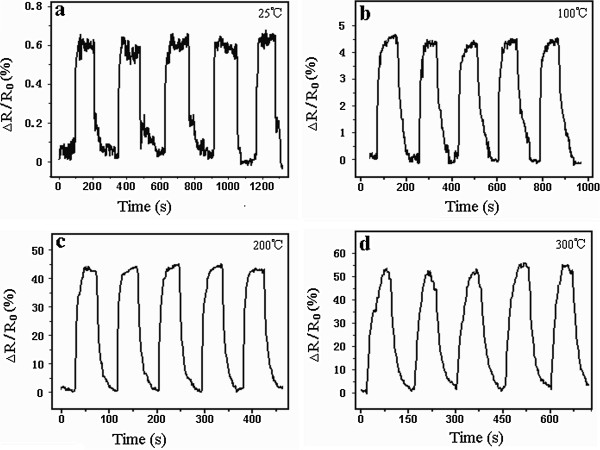
**Response curves of oxide nanofilms annealed at 550°C.** The operating temperatures were **(a)** 25°C, **(b)** 100°C, **(c)** 200°C, and **(d)** 300°C.

The oxide nanofilm annealed at 550°C had sensitivity for the 1,000 ppm H_2_ atmosphere at both room temperature and elevated temperatures. Figure 6 shows the response curves of the nanofilm sensor tested at temperatures ranging from 25°C to 300°C. The saturation response of the nanofilm sensor increased from around 0.6% at 25°C to more than 50% at 300°C, which also indicated that increasing the operating temperature will greatly enhance the hydrogen sensing performance of the Ti-Al-V-O nanofilm sensor. Unlike the nanofilm annealed at 450°C, the nanofilm annealed at 550°C demonstrated a quicker response and much stable sensing behavior by regaining its original resistance after air flushing in each testing cycle. The quick response of the Al- and V-doped nanofilm at 25°C was remarkable since undoped TiO_2_ nanotube sensors tested at room temperature usually had a minute-level response [24].

**Figure 7:**
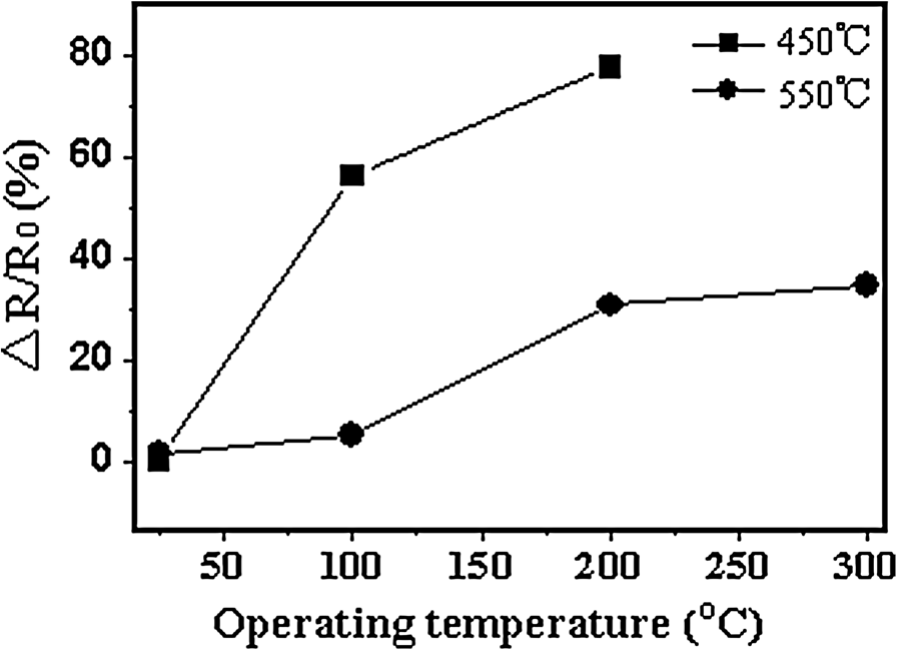
Saturation response of the oxide nanofilms to the 1,000 ppm hydrogen atmosphere.

As shown in Figure 7, overall response of the nanofilm annealed at 550°C shows advantage over those of the nanofilm annealed at 450°C. Based on the XRD analyses and the above sensing performance, it can be inferred that a higher annealing temperature could result in the formation of more anatase phases in the doped nanofilm. Larger quantity of anatase phases should enhance the adsorption and desorption of H_2_ molecules to the oxide nanofilm and thus enhance the hydrogen sensing performance.

## Discussion

Doping of TiO_2_ oxide with 1 to 5 mol% or 5% to 12% V element has been reported by Kahattha et al. and Hong et al. [25,26]. Also, Al-doped TiO_2_ oxide has been reported by Berger et al., Tsuchiya et al., and Nah [27-29]. The uniform doping of other elements in TiO_2_ oxide has been also reported in several literatures, including the report of lattice widening in Nb-doped TiO_2_ nanotubes [21,23,30]. According to our EDX point and area analyses, the Ti, Al, V, and O elements uniformly distributed in the analyzed area of the oxide layer. We did not find the aggregation of TiOx, AlOx, and VOx. This suggests that pure TiO_2_ oxide could not exist for our present oxide film. Although our XPS analyses could only indicate the chemical valence states of Al and V elements rather than proof for the Al and V doping in the lattice of TiO_2_ oxide, our XRD analyses revealed that the main diffraction peaks (25.28°, 48.38°, and 53.88°) of pure anatase TiO_2_ shifted to a certain degree due to the coexistence of Al and V elements. This indicated that the doping of Al and V elements into the TiO_2_ lattice could result in a shift of diffraction peaks of TiO_2_ oxide. Based on the above analyses, we believe that the present oxide film is a kind of Al- and V-doped TiO_2_ nanostructures.

In general, TiO_2_ nanotubes are n-type semiconductors by showing resistance decrease in reducing atmosphere like hydrogen and resistance increase in oxidizing atmosphere like oxygen. In our experiment, all of the as-annealed Ti-Al-V-O oxide nanofilms presented resistance increase upon exposure to the hydrogen atmosphere. This indicates that semiconducting characteristics of the TiO_2_ oxide here have been affected by doping with Al and V elements. A partial transformation from n-type semiconductor to p-type semiconductor may happen due to element doping. Through a modeling technique, Williams and Moseley theoretically predicted that conductance type of semiconducting oxides could change with the doping elements [31]. The following experiments proved that the semiconductor characteristics of TiO_2_ could change when doped with certain amounts of Cr [32], Nb [33], and Cu [34] elements. Liu et al. found that Nb doping did not alter the n-type hydrogen sensing behavior of anatase TiO_2_ nanotubes [23]. Moreover, it was found that TiO_2_ nanotubes could keep the n-type nature when doped with a certain amount of boron. Also, the nanotubes may keep n-type semiconductors or transform to p-type semiconductors when doped with nitrogen, depending on whether or not nearby oxygen atoms occupy surface sites [35].

There are many factors that could affect the hydrogen sensing performance of the Al- and V-doped TiO_2_ nanofilms. Nanotubular geometry, polymorph, element doping, and testing temperature affected the hydrogen sensing properties of the nanofilm sensors. Varghese et al. found that undoped TiO_2_ nanotubes with a smaller diameter (22 nm) could have a higher sensitivity for 1,000 ppm H_2_ at 290°C [36]. Anatase, the polymorph of TiO_2_, has been reported to be highly sensitive to reducing gases like hydrogen and carbon monoxide [37]. The hydrogen atom could diffuse to the interstitial sites of TiO_2_. As the c/a ratio of anatase phase is almost four times that of the rutile phase, the anatase TiO_2_ phase thus has a greater contribution to hydrogen sensitivity [7].

In the present oxide system, the nanofilms consisted of anatase phase favorable for hydrogen sensing at different temperatures. There are more defects and dislocations in the anatase structures than other crystalline structures [38,39]. Al and V atoms had an atomic radius different from Ti atom. Thus, Al and V doping could produce more lattice vacancy to capture electrons and accelerate the electron change which is beneficial for the chemical adsorption of hydrogen at the surface and therefore enhance the hydrogen sensitivity. Furthermore, an increased operating temperature of the nanofilm sensor could accelerate the diffusivity of the hydrogen atoms to the surface of the nanofilms and thus lead to a higher sensitivity.

As a ceramic oxide fabricated on robust metal substrate, the doped nanofilm provides a robust sensor unit working at either room temperature or elevated temperatures. The hydrogen sensing capability shown by the Al- and V-doped nanofilms makes it possible to further explore the semiconducting characteristics and hydrogen sensing behaviors of various kinds of TiO_2_ nanofilms with different dopant levels (i.e., Al/V ratio).

## Conclusions

In summary, Ti-Al-V-O oxide nanofilms with anatase structures were prepared by anodization and annealing. Annealing at different temperatures was found to result in different hydrogen sensing performances. Al and V doping was found to reduce the bandgap of TiO_2_ oxide. The Al- and V-doped anatase nanofilms demonstrated a p-type hydrogen sensing characteristics, which was quite different from the undoped TiO_2_ nanotubes. The Ti-Al-V-O nanofilms annealed at 450°C demonstrated sensitivity for 1,000 ppm H_2_ at elevated operating temperatures, while Ti-Al-V-O nanofilms annealed at 550°C had good sensing response at both room temperature and elevated temperatures.

## Competing interests

The authors declare that they have no competing interests.

## Authors' contributions

ZL participated in the experimental design, carried out the experiments, tested the thin films, and wrote the manuscript. DD and CN designed the experiments and testing methods, and helped draft the manuscript. All authors read and approved the final manuscript.
